# The Slr0058 Protein From *Synechocystis* sp. PCC 6803 Is a Novel Regulatory Protein Involved in PHB Granule Formation

**DOI:** 10.3389/fmicb.2020.00809

**Published:** 2020-04-30

**Authors:** Moritz Koch, Tim Orthwein, Janette T. Alford, Karl Forchhammer

**Affiliations:** Interfaculty Institute of Microbiology and Infection Medicine, Eberhard Karls Universität Tübingen, Tübingen, Germany

**Keywords:** PHB (poly-(3-hydroxybutyrate)), Cyanobacteria, bioplastic, Sustainability, *Synechocystis* 6803, phasin, PHB-depolymerase, biodegradability

## Abstract

During phases of nitrogen starvation, the photosynthetic cyanobacterium *Synechocystis* sp. PCC 6803 produces polyhydroxybutyrate (PHB). This polymer is of high biotechnological relevance because of its potential as biodegradable plastic. Analysis of the *Synechocystis* genome revealed an operon (*slr0058-slr0061*) containing several genes, which are putatively related to the PHB metabolism. While Slr0058 show similarities with the regulatory phasin PhaF, the protein Slr0060 could serve as an intracellular PHB depolymerase. Investigation of respective knock-out mutants showed no distinct phenotype for the strain lacking Slr0060, whereas the Δ*slr0058* mutant displayed a growth impairment as well as a change in pigmentation. During nitrogen starvation, the Δ*slr0058* mutant produced in average more than twice the amount of PHB granules per cell, while the overall amount of PHB remained the same. This indicates that Slr0058 plays a role in PHB granule formation and controls it surface-to-volume ratio. GFP-tagged Slr0058 did not co-localize with PHB granules during nitrogen starvation but aggregated in distinct foci during vegetative growth. This work helps to better understand the PHB metabolism of *Synechocystis* sp. PCC 6803, coming closer to a sustainable, industrial production of PHB.

## Introduction

Cyanobacteria are a diverse group of bacteria, which are able to perform oxygenic photosynthesis. Due to their phototrophic lifestyle they are considered as potential hosts for the sustainable production of industrially relevant compounds. The cyanobacterium *Synechocystis* sp. PCC 6803 (hereafter *Synechocystis*) is one of the best-studied cyanobacterial model strains (Yu et al., 2013). Its fully sequenced genome and the availability of various genetical tools make *Synechocystis* to a versatile microbial chassis (Kaneko et al., 1995; Liu and Pakrasi, 2018). Besides that, *Synechocystis* is also an important strain for basic research, due to its diverse lifestyles, as it supports phototrophic, heterotrophic or mixotrophic growth (Zavřel et al., 2017). Depending on the growth conditions, *Synechocystis* evolved to produce numerous biopolymers, such as polyphosphate, cyanophycin glycogen or polyhydroxybutyrate (PHB) (Damrow et al., 2016). The last two are particularly relevant under the conditions of nitrogen starvation. For this condition, *Synechocystis* has evolved a unique adaptation strategy, namely chlorosis (Collier and Grossman, 1992; Krasikov et al., 2012). During this highly orchestrated process, the cells undergo a transformation into a resting state, allowing the cells to survive prolonged periods of nitrogen starvation (Schwarz and Forchhammer, 2005). As defined for *Synechococcus elongatus*, chlorosis can be divided into several successive steps (Görl et al., 1998). Immediately upon nitrogen depletion, the cells start to accumulate large quantities of glycogen and they rapidly degrade the major antenna proteins, the phycobiliproteins. Subsequently, in a second phase, the photosynthetic apparatus with chlorophyll *a* is degraded until only traces of photosynthetic activity remain. Finally, the cells enter a state of metabolic quiescence in which they remain viably for prolonged periods of time. In principle, the same process occurs in *Synechocystis*, with the main difference that *Synechocystis* gradually accumulates PHB by glycogen turnover in the second phase of chlorosis (Koch et al., 2019). Upon the addition of nitrogen, the chlorotic process is reversed: in a two-phase event, cells mobilize glycogen to restore their photosynthetic machinery and then switch to phototrophy (Klotz et al., 2016; Doello et al., 2018).

Although *Synechocystis* is a well-studied model strain, many aspects of PHB granule formation and metabolism remain unknown. Until today, the physiological function remains puzzling (Damrow et al., 2016) and only few studies have characterized the physical properties of PHB (Osanai et al., 2013; Troschl et al., 2017). Additionally, many essential components yet have to be discovered, like a PHB degrading depolymerase. So far, only one PHB granule associated protein has been identified, the phasin PhaP, which appears to be involved in controlling the upper size of PHB granules (Hauf et al., 2015). It is known from other PHB producing bacteria that the PHB metabolism is a complex process, where many different proteins are involved. Due to its complexity, the term “carbonosom” was suggested for PHB granules (Jendrossek, 2009). The model strains *Cupriavidus necator* and *Pseudomonas putida* harbor a set of different proteins which are involved in the PHB metabolism, often referred to as phasins. Among them are PHB polymerases (PhaC) and depolymerases (PhaZ and PhaY), transcriptional regulators (PhaI), or other regulatory phasins such as PhaF and PhaM (Pfeiffer and Jendrossek, 2011). Preliminary work in our lab indicated that in an operon of so far uncharacterized proteins, novel PHB associated proteins may be present. This work aims to characterize the two putative phasins Slr0058 and Slr0060 and their role for the PHB metabolism. The knowledge could help to further improve the synthesis of PHB in cyanobacteria for a sustainable production of bioplastics.

## Materials and Methods

All experiments shown in this work are based on three biological replicates unless stated otherwise. The arithmetic mean was calculated including the standard deviation, as indicated by the error bars at each measurement point.

### Cyanobacterial Cultivation Conditions

For preculturing and growth experiments, *Synechocystis* was cultivated in BG_11_ medium (Rippka et al., 1979). All cultivations with *Synechocystis* were performed in baffle free Erlenmeyer shaking flasks. Standard cultivation was performed at 28°C with 125 rpm shaking and continuous illumination of ∼50 μE m^–2^ s^–1^ unless stated otherwise. Aeration was ensured by continuous shaking and ambient air. No additional gassing with CO_2_ was applied. Either 50 or 200 ml of cell suspension were cultivated in 100 or 500 ml Erlenmayer flasks, respectively. Whenever necessary, appropriate antibiotics were added to the different strains to ensure the continuity of the mutation.

Chlorosis was induced by changing the medium to nitrogen-depleted BG_0_, which contains the same components as BG_11_, except sodium nitrate. For the medium change, *Synechocystis* cultures were precultivated in BG_11_ medium up to an OD_750_ of ∼0.8. The cells were washed with BG_0_. For this, the cells were centrifuged, resuspended in BG_0_ medium and adjusted to OD_750_ of 0.4. Cells were resuscitated from nitrogen starvation by exchanging the growth medium to standard BG_11_.

For viability assays, BG_11_ plates containing 1.5% agar were used. Cultures of OD_750_ = 0.4 (10^0^) were diluted to 10^–1^, 10^–2^, 10^–3^, and 10^–4^. Of each dilution, 5 μl were dropped on a BG_11_ agar plate. The plates were cultivated seven days at either continuous light or day-night conditions (12 h light and 12 h darkness).

A list of all used strains for this study is provided in Supplementary Table S1.

### Molecular Cloning and Transformation Into Synechocystis

For the assembly of DNA, Gibson cloning was used (Gibson et al., 2009). Lists of all used primers and plasmids of this study are provided in Supplementary Tables S2, S3. Cultivation of *Escherichia coli* was done as previously described (Sambrook, 2001). Successful cloning was verified via sequencing. Transformation into *Synechocystis* cells were done via natural transformation in the case of insertions in the genome. Successful segregation was checked via colony PCR. Whenever transient plasmids were introduced, triparental mating was used.

### Microscopy and Staining Procedures

The visualization of PHB granules was achieved via Nile red staining and subsequent analysis using fluorescent microscopy. For this purpose, the Leica DM5500 B with the Leica CTR 5500 illuminator was used. The integrated Leica DFC 360 FX camera was used for image acquisition. Settings were adjusted by the Leica Application Suite Advanced Fluorescence (4.0). For staining of PHB granules, 100 μl *Synechocystis* culture was centrifuged at 16.000 × *g* for 1 min. The supernatant (80 μl) was discarded and the pellet was resuspended 10 μl Nile red solution (1 μg/ml). From this mixture, 10 μl were dropped on an agarose coated microscope slide and were observed with 1000-fold magnification using the Leica HCX PL FLUATAR (100x/1,30 PH3) with immersion oil. The fluorescence of Nile red was induced with light of 558 nm wavelength (Cy3 channel). For electron microscopy pictures, *Synechocystis* cells were stained using glutaraldehyde and potassium permanganate. Subsequently, ultrathin sections were stained with citrate and uranyl acetate, as described before (Fiedler et al., 1998). The samples were then examined, using a Philips Tecnai 10 electron microscope at 80 kHz.

### PHB Quantification

Polyhydroxybutyrate was quantified by high-performance liquid chromatography (HPLC) of crotonic acid. For this, a volume of 11 ml chlorotic culture (or ∼25 ml of vegetative culture) was centrifuged at 4,000 × *g* for 10 min at room temperature. The supernatant was discarded, and the pellet transferred into a pre-balanced 2 ml reaction tube. Subsequently the pellet was dried with a vacuum dryer. The reaction tube was cradled again and the cell dry weight (CDW) of the culture was calculated. The cells were boiled for 1 h in concentrated H_2_SO_4_ (18 M). During this treatment, the cells were lysed and PHB converted to crotonic acid. Next, 110 μl of this solution was diluted 1:10 with 14 mM sulfuric acid solution and centrifuged 5 min at 25,000 × *g*. Subsequently, 500 μl of the clear supernatant was mixed with 500 μl 14 mM H_2_SO_4_, pelleted again and 300 μl of the supernatant was used for analytical HPLC. For this, a HP1090 M chromatography system was used, equipped with a thermostated autosampler und diode-array-detector, HP Kayak XM 600 workstation. The crotonic acid was detected by measuring the absorbance at 210 nm.

### Glycogen Quantification

The glycogen content of *Synechocystis* cultures was detected as described previously (Grundel et al., 2012) with modifications described (Klotz et al., 2016). In short, 2 ml of cell culture were centrifuged for 1 min at 16,000 × *g*. The supernatant was discarded, and the pelleted cells were used for the measurement. Degradation of glycogen was performed with 4,4 U/μl amyloglucosidase from *Aspergillus* (Sigma Aldrich) for 2 h at 60°C. The concentration of resulting glucose units was measured with an o-toluidine assay at a wavelength at 635 nm. The glycogen concentration was determined by comparison with a glucose standard calibration curve.

### Spectra and Pigment Determination

The absorption spectra between 350 and 750 nm of *Synechocystis* strains were detected with a Specord50 and the software WinAspect (Jena Analytik). The content of chlorophyll *a* (Chl *a*) in cells was detected with a methanol extraction method. For this, 1.5 ml of an exponential culture was transferred into a 1.5 ml reaction tube and centrifuged at 10,000 × *g* for 10 min. The supernatant was discarded, and the pellet was resuspended in 90% Methanol. The suspension was mixed and subsequently incubated for 30 min in the dark. The suspension was centrifuged again and the supernatant was transferred into a 1.5 ml reaction tube. Another 500 μl 90% methanol were added to the cell pellet and treated as described above. Both supernatants were combined and transferred into a cuvette. The absorbance was measured at wavelengths of 665 and 650 nm, and the Chl *a* concentration was calculated according to the equation previously described (Richmond and Hu, 2013).

### Pulse-Amplitude-Modulation Fluorometry

The pulse-amplitude-modulation fluorometry (PAM) was used to detect the photosynthetic activity by measuring the relative quantum yield of photosystem (PS) II of the photosynthetic apparatus. For this, the WATER-PAM Chlorophyll Fluorometer with WinControl Software of Heinz Walz GmbH (Effeltrich) was used. In the measuring cuvette, cell suspensions between OD_750_ = 0.4 – 1 were diluted 1:20. The cuvette was placed in the measuring device and the yield was measured three times with a time constant of 30 s.

### Overexpression and Purification of Slr0058

*Escherichia coli* Lemo21 (DE3) (NEB) containing the plasmid pET15b-slr0058-His was grown overnight at 37°C in 5 ml LB-medium containing 50 μg/ml ampicillin and 30 μg/ml chloramphenicol. The Cells were transferred into 1 l of fresh LB-medium, containing the same antibiotics and growth was continued at 37°C to an OD_600_ of 0.6. Slr0058 overexpression was induced by the addition of 0.5 mM IPTG to the culture, which was subsequently grown overnight at 20°C. Cells were harvested by centrifugation at 6500 rpm for 10 min at 4°C. The resulting pellet was resuspended in buffer containing 300 mM NaCl, 50 mM NaH_2_PO_4_, 10 mM imidazole, 1 mM DTT, 0.2 mM PMSF, DNase I and was adjusted to pH 8. The cells were lysed by four cycles of ultrasonication (Branson Ultrasonics^TM^), with a sonication time of 3 min per cycle. Remaining cells and cell debris were removed by centrifugation at 10000 rpm for 60 min at 4°C and subsequently filtering the resulting supernatant through a 0.45 μm syringe filter. The supernatant was loaded on a 1 mL HisTrap FF Ni-NTA column (GE Healthcare), using a flowrate of 1 ml/min. The column was washed with buffer (pH 8) containing 300 mM NaCl, 50 mM NaH_2_PO_4_, and 20 mM imidazole followed by a washing step with an imidazole concentration of 40 mM to remove unspecifically bound protein. Finally, the His-tagged protein was eluted by increasing the imidazole concentration to 250 mM. The eluted protein was transferred to a buffer containing 100 mM KCl, 50 mM Tris–HCl, pH 7.8, 5 mM MgCl_2_, 1 mM DTT, 0.5 mM EDTA and 50% (v/v) glycerol via dialysis overnight at 4°C and then stored at −20°C. The eluted protein, as well as samples from each purification step were analyzed by SDS-PAGE.

### Size Exclusion Chromatography

To investigate the size and oligomerization states of Slr0058, size exclusion chromatography was performed on a ÄKTAmicro (GE Healthcare) using a Superose^TM^ 6 Increase 10/300 GL column (GE Healthcare). The column was equilibrated with a buffer containing 100 mM KCl, 50 mM Tris–HCl, pH 7.8 and 5 mM MgCl_2_. The Slr0058 protein was transferred into the same buffer and a volume of 100 μl with a concentration of 0.313 mg/ml was used for the size exclusion chromatography. Data were analyzed with UNICORN 5.20.

## Results

**FIGURE 1:**
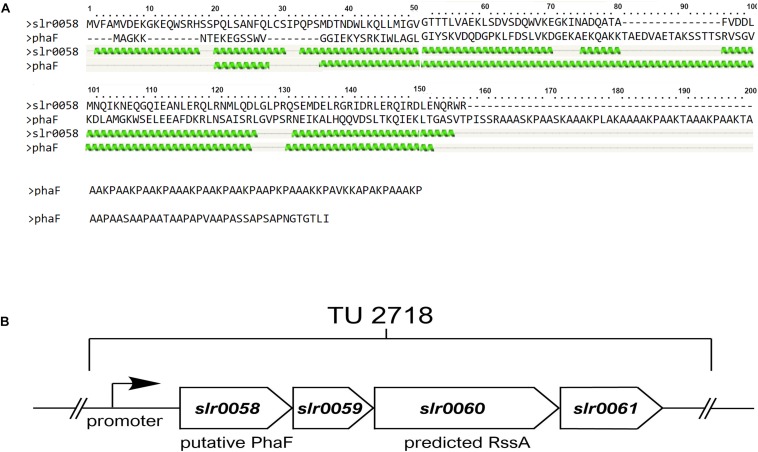
*In silico* analysis of a PHB related gene cluster. **(A)** Sequence allignment and the predicted structure of PhaF from *Pseudomonas putida* and Slr0058 from *Synechocystis*. Both proteins show regular pattern of helix structures at the N-terminus (indicated as green helices). Unlike PhaF, Slr0058 shows no prolonged C-terminal coil structure though, which is important for the interaction with the DNA in *Pseudomonas putida*. **(B)** Schema of the transcriptional unit 2718 in *Synechocystis.* The operon contains four genes, *slr0058*–*slr0061*. Two of them, namely *slr0058* and *slr0060*, have sequence similarities with either PhaF, or a PHB depolymerase, respectively.

By analyzing the *Synechocystis* genome for putative homologies of phasins, we identified an operon of genes of unknown function, containing members that show similarities to phasins from other organisms. The transcriptional unit TU2718 consists of four genes, namely *slr0058* – *slr0061*. Transcriptome analysis revealed that the entire operon is upregulated during chlorosis (Mitschke et al., 2011; Klotz et al., 2016). For one of these proteins, a BLAST analysis revealed that the protein Slr0058 has similarity with the protein PhaF of *Pseudomonas putida*. This protein ensures an equal distribution of the PHB granules among the daughter cells during cell division by attaching the granules to the chromosome (Maestro et al., 2013). Unlike PhaF from *P. putida* Slr0058 showed no DNA binding domain though (Figure 1).

Another gene in this operon, *slr0060*, encodes a protein, which was previously isolated during the purification of PHB granules. Subsequent bioinformatic analysis showed that Slr0060 was only found in cyanobacteria known to produce PHB (Schlebusch, 2012). A recent proteome study revealed distinct compartment organization of the *Synechocystis* proteins. Here Slr0060 was found in a distinct group together with other PHB related proteins, PhaP, PhaE and PhaC, further supporting its relevance for PHB metabolism (Baers et al., 2019). BLAST search of Slr0060 predicts a patatin domain, which was also found in phospholipases/acylesterases. This indicates that Slr0060 might serve as an intracellular PHB depolymerase (1 B).

To characterize the function of *slr0058* and *slr0060*, knock-out strains were constructed (pMK17 and pMK16, respectively). For this, the genes were replaced by an antibiotic-cassette using double homologous recombination. Plasmids containing DNA fragments, which are flanked by homologous sequences of *slr0058* or *slr0060*, were transferred into *Synechocystis* by natural transformation. To prevent any unintended effects on genes located downstream of the deleted gene, the native promoter was introduced downstream of the antibiotic cassettes. The structure of the plasmids is shown in Supplementary Figure S1.

The construction of pMK16 and pMK17 was performed using Gibson Assembly. The vector backbone pUC19 was linearized using the restriction enzymes *Xba*I and *Pst*I. Successful cloning was verified by colony PCR and subsequent sequencing. From now on, *Synechocystis* mutants transformed with pMK16 are referred to as Δ*slr0060* and mutants edited with pMK17 as Δ*slr0058*.

### Growth Behavior of Δ*slr0058* and Δ*slr0060* Strains

**FIGURE 2:**
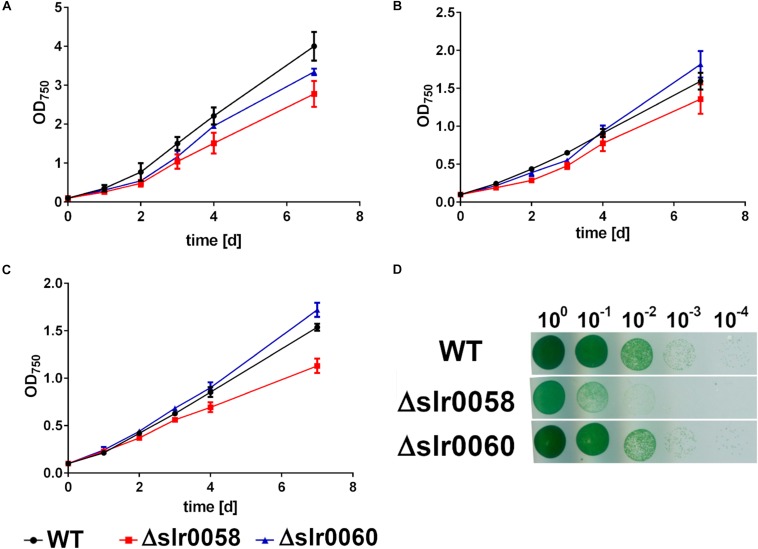
Growth behavior of WT, Δ*slr0058*, and Δ*slr0060* at different cultivation conditions. **(A)** Cultivation under continuous illumination and with shaking. **(B)** Cultivation with 12 h day/night cycle and shaking. **(C)** Cultivation with 12 h day/night cycle and without shaking. Each point represents a mean of three independent biological replicates. **(D)** Growth determination using the drop plate method of 10-fold dilutions at continuous light cultivation. The plate shown in the figure is representative for three individually grown biological replicates.

To characterize the created mutant strains Δ*slr0058* and Δ*slr0060*, their growth in liquid medium under various culturing conditions and on solid agar plates was compared to a wildtype strain (WT) (Figure 2).

To characterize the growth in liquid medium, several combinations of illumination and shaking were tested. Besides standard laboratory conditions, where the cells were shaken under continuous illumination, they were also grown under naturally occurring day-night rhythm. Here they were either shaken or grown under standing conditions resulting in reduced aeration (Figures 2A–C, respectively). Cell growth was observed by measuring OD_750_ every 24 h of three independent biological replicates. Under all conditions, the strains WT and Δ*slr0060* showed similar growth. In contrast, the strain Δ*slr0058* showed a slight growth deficiency under all tested conditions. The strongest effect was visible during day-night rhythm with standing cultures (Figure 2C). The growth deficiency was even more clearly visible during growth on solid agar plates (Figure 2D).

**FIGURE 3:**
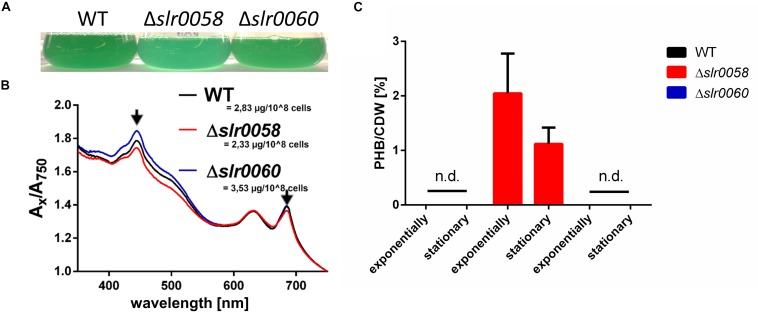
Phenotypical analysis of WT, Δ*slr0058*, and Δ*slr0060* during vegetative growth. **(A)** Picture of representative WT, Δ*slr0058* and Δ*slr0060* cultures, at OD_750_ = 0.8. **(B)** Spectra and chlorophyll *a* concentrations of the same cultures as in panel **(A)**. **(C)** PHB produced during vegetative growth. Samples were taken during exponential or stationary growth phases (OD_750_ ∼1 or ∼4, respectively). The values measured for the WT and Δ*slr0060* were below to the detection limit. n.d. = not detectable.

Besides the different growth characteristics, the strains showed also a difference in their pigmentation. While only little differences were visible between the color of WT and Δ*slr0060* strains, the Δ*slr0058* culture appeared more bluish (Figure 3A).

Spectral analysis revealed that a slightly reduced absorption at ∼430 nm for the strain Δ*slr0058*, indicating a lower abundancy of Chl *a* content (Figure 3B). This correlates well with the measured chlorophyll content, which was reduced in the strain Δ*slr0058* compared to the WT (2.33 to 2.83 μg/10^8^ cells, respectively). While the strain Δ*slr0060* showed higher values of chlorophyll content (3.53 μg/10^8^ cells), no strong difference was visible in the appearance of the culture. During vegetative growth, the amount of PHB produced in the WT and Δ*slr0060* were below the detection limit (Figure 3C). In contrast, the mutant strain Δ*slr0058* produced detectable amounts of PHB during exponential and stationary growth (∼2 and ∼1%/CDW, respectively) under diurnal light/dark regime. Despite the presence of considerable amounts of PHB, no fluorescent granules could be observed by microscopy after staining the cells with BODIPY. This indicates that PHB produced during vegetative growth is not aggregated into stainable PHB granules. When grown under continuous light, there was no PHB detected in any of the strains.

### Mutant Phenotype During Chlorosis

**FIGURE 4:**
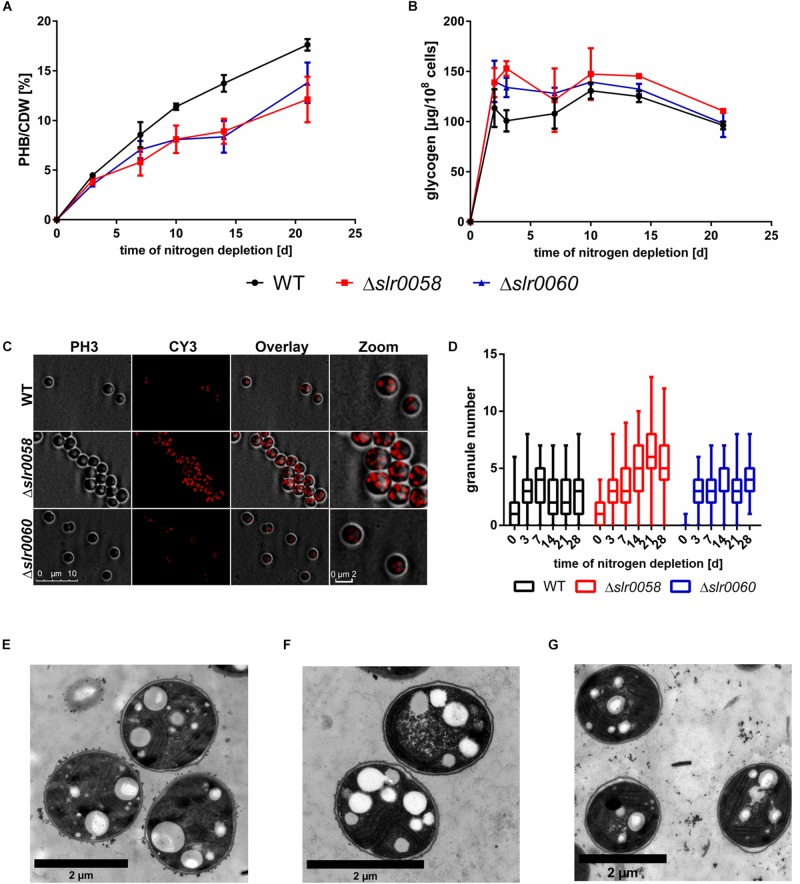
Phenotypical analysis of WT, Δ*slr0058*, and Δ*slr0060* during chlorosis. **(A)** Accumulation of PHB per cell dry weight (CDW) during 21 days of chlorosis. **(B)** Accumulation of glycogen normalized to OD_750_ during 21 days of chlorosis. Each point represents a mean of three independent biological replicates. **(C)** 3-D deconvoluted microscopic images of 21-day nitrogen depleted cultures of all three strains. The cells were stained with Nile red to visualize PHB granules. From left to right: image of the phase contrast channel; image of the Cy3 channel with Nile-red stained PHB granules; overlay of both channels. **(D)** Box plot of the granule number at different time points of chlorosis. Each time point represents the count of 70 cells in three independent cultures. The line within the boxes represents the median. **(E–G)** Electron microscopic pictures of WT **(E)**, Δ*slr0058* **(F)**, and Δ*slr0060* **(G)** cells after 17 days of nitrogen starvation. PHB granules are visible as white inclusions inside the cell.

To determine the effect of the absence of Slr0058 and Slr0060 during conditions where PHB is formed, chlorosis was induced by shifting the cultures to nitrogen-depleted medium. As previously reported from our laboratory, PHB is mainly formed from glycogen. Hence, PHB and glycogen were measured during the course of 28 days (Figures 4A,B).

In all three strains the intracellular PHB increased over time. As compared to the WT, both mutant strains showed reduced amounts of PHB during the course of the experiment. Like in previous studies, the cells rapidly accumulated glycogen during the initial phase of the chlorosis, to a maximum level which then slowly declined. Although the overall pattern was similar. the two mutant strains showed slightly elevated glycogen values compared to the WT. The OD_750_ of the WT and Δ*slr0060* were comparable and remained constant during the course of chlorosis. In contrast, the OD_750_ of Δ*slr0058* constantly decreased during the 4 weeks of the experiment (Supplementary Figure S2A). This decrease indicates a decrease in cell number, suggesting that the Δ*slr0058* mutant is impaired in maintaining long-term viability.

To estimate size and number of PHB granules, cells in chlorosis for 21 days were stained with Nile red and analyzed using fluorescence microscopy (Figures 4C,D).

The strain Δ*slr0058* showed a strong increase in the amount of PHB granules compared to the WT and the Δ*slr0060* strain (Figure 4C). To quantify this, the visible granules from 70 cells in three biological replicates were counted and plotted in a box blot (Figure 4D). The granule number was determined within 28 days of nitrogen depletion. The analysis confirmed the visible effect of the microscopic pictures of an altered granule number within the strains. The WT and Δ*slr0060* showed very similar amounts of PHB granules. Compared to this, Δ*slr0058* showed a more than 2-fold increase in the number of granules. This observation was also confirmed by electron microscopic pictures (Figures 4E–G).

As already indicated by fluorescence microscopy (Figure 4C), the Δ*slr0058* mutant cells (Figure 4F) contained a strongly increased number of PHB granules compared to the WT and Δ*slr0060* cells (Figures 4E,G, respectively).

**FIGURE 5:**
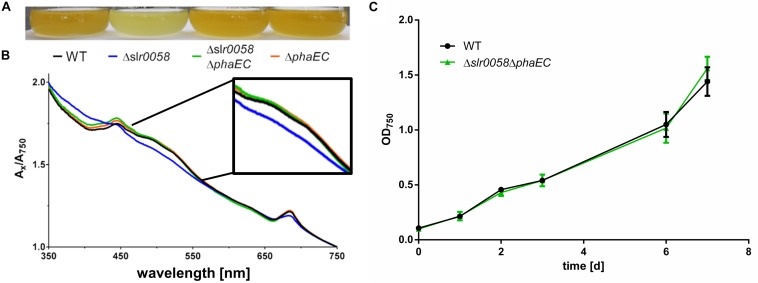
Photograph **(A)** and spectra **(B)** of WT, Δ*slr0058* and Δ*slr0060* cultures during chlorosis compared with a PHB free mutants (Δ*phaEC*). The spectra of the different strains were normalized to OD_750_ = 1. **(C)** Growth behavior of WT and Δ*slr0058*Δ*phaEC* with 12 h day/night cycle and without shaking.

Similar than during exponential growth, the cultures showed a difference in their pigmentation (Figure 5A). Compared to the WT, the culture was less pigmented, indicating a reduction in the carotenoid content. This assumption was confirmed by a spectral analysis of the cultures, where a lower absorption between 450 and 550 nm is visible (Figure 5B).

To check if the phenotypes observed for the Δ*slr0058* strain, in particular the growth impairment and altered pigmentation are related to the synthesis of PHB, a mutant lacking the PHB synthase was created by deleting the *phaEC* operon (Δ*phaEC* strain). To validate how the absence of PHB synthesis affects the phenotype of the Δ*slr0058* strain, both deletions were combined in one strain (Δ*slr0058* and Δ*phaEC*). As shown in Figure 5, the deletion of *phaEC* complemented the phenotype of the Δ*slr0058* strain by restoring the original color (Figures 5A,B). The double mutant Δ*slr0058*Δ*phaEC* showed also no difference in its growth behavior compared to the WT (Figure 5C). This suggests that the phenotype of the Δ*slr0058* strain is caused by the formation of PHB in the absence of Slr0058 protein.

### Resuscitation From Nitrogen Chlorosis

**FIGURE 6:**
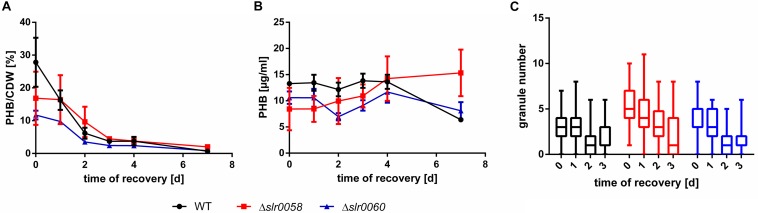
PHB amount and granule number in WT, Δ*slr0058* and Δ*slr0060* during resuscitation from 14-day chlorosis. **(A)** PHB amount normalized to the cell dry weight and **(B)** absolute amounts per volume. Each point represents a mean of three independent samples. CDW = cell-dry-weight. **(C)** Box plot of the granule number within the first 3 days of resuscitation. Each time point represents the count of >100 cells in three independent cultures. The line within the boxes represents the median.

A putative PHB depolymerase is expected to be most active during conditions of PHB degradation. Since such conditions should prevail during resuscitation from nitrogen chlorosis, recovery experiments of long-term nitrogen-starved cells were performed. A mutant lacking PHB depolymerase should be unable to degrade PHB during the recovery from chlorosis. Furthermore, such experiments could reveal differences between different strains in their efficiency to recover from nitrogen starvation. The experiments were performed with 14-day chlorotic cultures. The levels of PHB were determined every 24 h for four consecutive days and a final measurement after 1 week. The amount of PHB was normalized against the cell-dry-weight (CDW) as well as against the culture volume (to consider the increase of CDW due to cell growth). Before and after each sampling, the cultures were weighted, and the evaporated volume was restored with sterile ultrapure water. The results of PHB during recovery are shown in Figure 6.

When normalized to the CDW, the amount of PHB per CDW in all three strains decreased steadily during the course of 5 days, as already shown previously (Figure 6A). In contrast, when the absolute quantity of PHB per culture volume was determined, a surprising different pattern appeared. The total amount of PHB remained constant in all three strains during the entire resuscitation process (Figure 6B). Since PHB is usually normalized to the CDW, this observation has not been made before. While the overall amount of PHB remained constant, the abundance of granules declined in all three strains ($\bar{X}$_WT,__0__d_ = 2.8 ± 1.6, $\bar{X}$_WT,__3__d_ = 1.7 ± 1.4; $\bar{X}$_Δ_*_*slr*__0058_*,_0__d_ = 5.5 ± 2.0, $\bar{X}$_Δ_*_*slr*__0058_*,_3__d_ = 2.4 ± 2.7; $\bar{X}$_Δ_*_*slr*__0060_*,_0__d_ = 3.7 ± 1.7, $\bar{X}$_Δ_*_*slr*__0060_*,_3__d_ = 1.4 ± 1.1) (Figure 6C).

**FIGURE 7:**
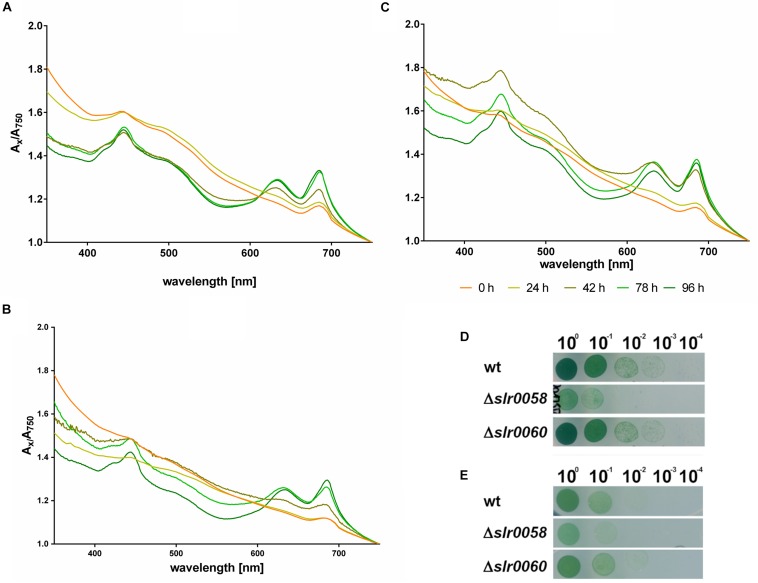
Pigment synthesis during resuscitation and viability after 14 days of chlorosis. Whole cell spectra from 350 to 750 nm of WT **(A)**, Δ*slr0058* **(B)** and Δ*slr0060* **(C)** after 0–4 days of resuscitation from nitrogen starvation. Each line represents the mean of the spectra from three independent biological triplicates. The spectra were normalized to their corresponding value at 750 nm. **(D)** Viability assays via drop-plate method as visible after 7 days of incubation under continuous illumination **(E)** and day-night illumination after 14 days of chlorosis. The images show a representative assay of triplicates.

To test whether the mutants were affected in their pigmentation during the recovery from nitrogen starvation, spectra measurements were performed. Additionally, the same cultures were used to test their viability after chlorosis via a drop-plate assay, shown in Figure 7.

The whole cell spectrum of WT cells at the beginning of the resuscitation shows the typical wavelength-dependent light scattering of low-pigmented cells (Figure 7A). After 2 days of recovery, distinctive absorption peaks for Chl *a* and phycobiliproteins appear at 430, 625, and 680 nm. The same appearance of absorption peaks was observed in the mutant strains Δ*slr0058* and Δ*slr0060* (Figures 7B,C). However both strains showed a visible delay. Particularly Δ*slr0060* required 2 days more to reach the same absorption in the range from 350 to 400 nm as the WT.

The recovery assays via the drop-plate method performed under continuous light or day-night rhythm (Figures 7D,E, respectively) revealed an impairment of the Δ*slr0058* strain. This impairment of growth for Δ*slr0058* was also visible when chlorotic cells resuscitated in liquid culture (Supplementary Figure S2B). However, the impairment of growth is in the same order of magnitude as during normal growth, indicating that this effect can be explained by a growth delay and not a deficiency during the recovery process. During the resuscitation, no difference in the recovery rate of the photosynthetic activity was visible (Supplementary Figure S3).

### Complementation of Δ*slr0058* and Intracellular Localization of Slr0058

The results of the Δ*slr0058* characterization showed that the Δ*slr0058* phenotypes are connected to the formation of PHB. To validate that the phenotype can be traced back to the deleted gene, a complementation strain of the gene *slr0058* was created (*slr0058*-pMK23). The latter is expected to restore the WT-phenotype. Additionally, the expression of a green fluorescence protein (GFP) tagged to Slr0058 (pMK25) should reveal its localization via fluorescence microscopy. The design of both constructs is shown in Supplementary Figure S4.

**FIGURE 8:**
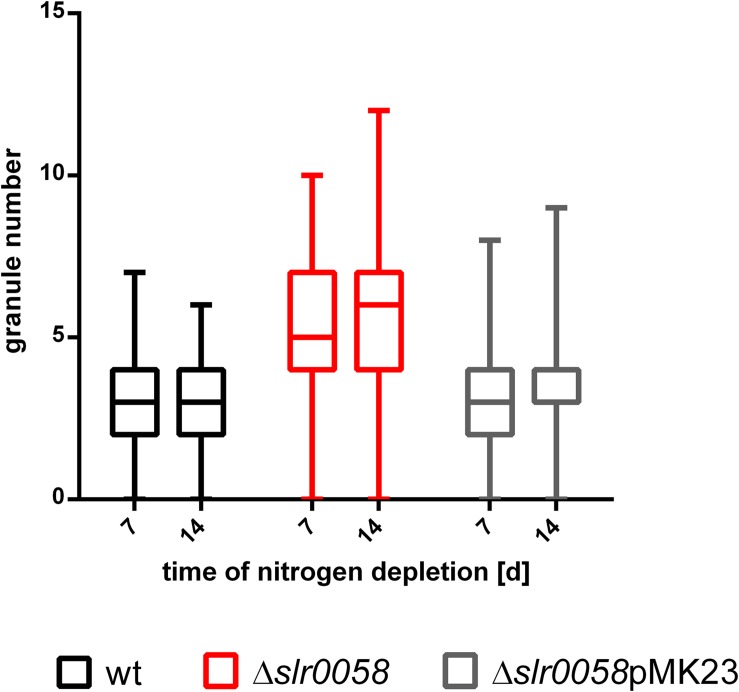
Box plot of the granule number at different time points of chlorosis of WT, Δ*slr0058* and complemented Δ*slr0058*pMK23. Each time point represents the result of a count of 150 cells in three independent cultures.

The assembly of the plasmids was done via Gibson Assembly. After transformation into *Synechocystis* cells, positive clones were screened via colony PCR. To ensure a successful complementation, the PHB granules were counted after one and 2 weeks in the WT, the *slr0058* and the complemented *slr0058*-pMK23 strain (Figure 8).

As expected, the insertion of pMK23 into the background of Δ*slr0058* restored the WT-phenotype, with respect to the average PHB granule number per cell. The WT and the Δ*slr0058*pMK23 complemented strain contained around three granules per cell on average ($\bar{X}$_WT,__7__d_ = 3.2 ± 0.1, $\bar{X}$_WT,__14__d_ = 3.1 ± 0.1; $\bar{X}$_Δ_*_*slr*__0058_*_pMK__23_,_7__d_ = 3.4 ± 0.1, $\bar{X}$_Δ_*_*slr*__0058_*_pMK__23_,_14__d_ = 3.2 ± 0.1). Compared to this, the average amount of granules in the strain Δ*slr0058* were almost 2-fold higher compared to the WT and the complemented strain ($\bar{X}$_Δ_*_*slr*__0058_*,_7__d_ = 5.5 ± 0.2, $\bar{X}$_Δ_*_*slr*__0058_*,_14__d_ = 5.7 ± 0.2). The results show that the effect of the elevated number of PHB granules is caused by the absence of Slr0058.

**FIGURE 9:**
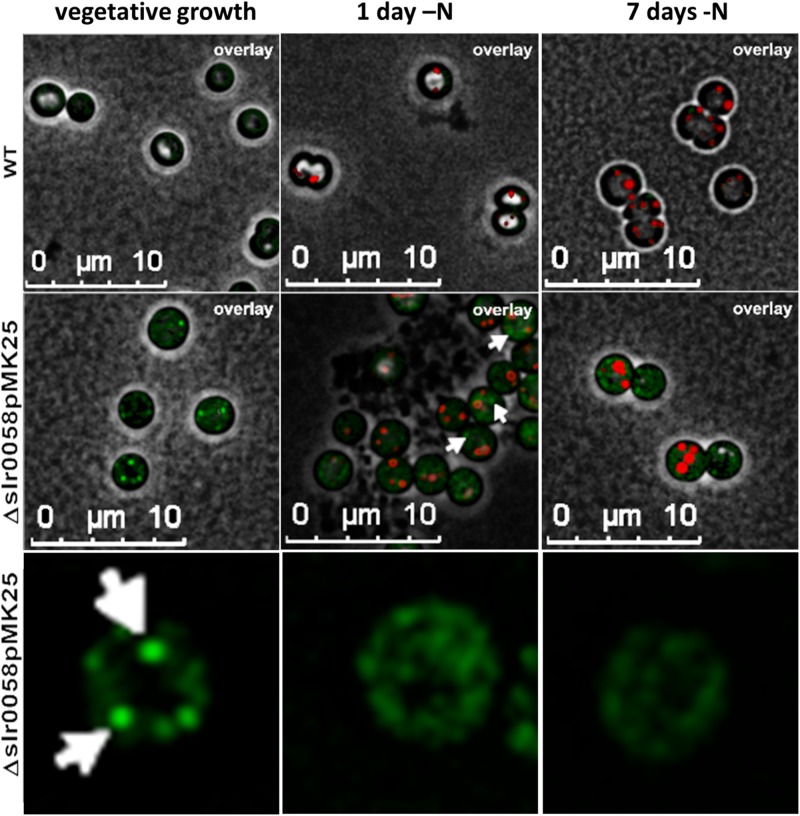
3-D deconvoluted microscopic images of WT and GFP-tagged-Slr0058 in the background of Δ*slr0058* (Δ*slr0058*pMK25) during vegetative growth and after 1 and 7 days of nitrogen starvation. PHB granules were stained with Nile red. White arrows indicate foci of aggregated GFP signal. The last row shows a zoom to individual, representative Δ*slr0058*pMK25 cells, in the GFP channel.

To reveal the subcellular localization of Slr0058, an eGFP-tagged Slr0058 variant (pMK25) was inserted into the Δ*slr0058* background. The localization of Slr0058 was observed under different growth conditions using fluorescence microscopy with a GFP channel for the eGFP excitation. WT and pMK25 cells were observed during vegetative growth and after a shift to nitrogen depletion. Microscopic pictures of these conditions are shown in Figure 9.

During vegetative growth, a low fluorescence signal in the GFP channel was visible in the WT cells, which could be caused by bleed-through effects of autofluorescence of Chl *a*. In comparison, a much higher fluorescence signal was detected in the strain pMK25, often in specific foci. These spots of strong fluorescence indicate focal aggregation of Slr0058 in the cytoplasm. After the induction of chlorosis, these spots started to disappear. No colocalization of the GFP-Slr0058 foci with Nile red stained PHB granules could be seen (white arrows, Figure 9). During the course of chlorosis, the GFP aggregates further disappeared. In agreement with the *in vivo* formation of foci, recombinant purified His-tagged Sll0058 protein, analyzed by size-exclusion chromatography, displayed different aggregation states (Supplementary Figure S5).

## Discussion

### Characterization of *slr0060* and Its Role for PHB Degradation

The gene product of *slr0060* was recently located in the same subcellular region as other PHB-related proteins, indicating a relevance of Slr0060 for the PHB metabolism (Baers et al., 2019). Furthermore, its sequence-homology to esterases suggests a role as a putative intracellular PHB depolymerase. However, a knock-out strain showed only minor difference to the WT in its growth during vegetative growth, chlorosis or resuscitation (Figure 2 and Supplementary Figure S2). The biggest difference compared to the WT was an increased chlorophyll content (Figure 3), as well as a reduced amount of produced PHB (Figure 4A). Furthermore, a slightly different whole cell light scattering was detected during resuscitation from chlorosis. However, the deletion of a putative PHB depolymerase is expected to cause enhanced levels of PHB or larger granules than the WT, particularly during the recovery process, which was not the case (Figure 6). Hence, the true function of *slr0060* remains unknown. One explanation for the missing phenotype could be a second, so far undiscovered PHB depolymerase homolog, which compensates the loss of Slr0060. *Synechocystis* is known for possessing two homologs sets of enzymes for central reactions in carbohydrate metabolism, such as paralog pairs of GlgA1/2, GlgP1/2, or GlgX1/2 (Doello et al., 2018; Koch et al., 2019). Accordingly, this could also be the case for another, yet undiscovered PHB depolymerase.

Interestingly, our analysis revealed that the total amount of PHB polymers is not diminished during resuscitation. This phenomenon was not detected before, since earlier studies normalized the amount of PHB to the CDW (as shown in Figure 6A; Klotz et al., 2016). However, this normalization is biased by the fact that during resuscitation, the CDW increases strongly. This raises the hypothesis that the disappearance of PHB granules, that is observed during recovery from nitrogen chlorosis, is primarily not the result of PHB depolymerization, but rather results from disaggregation and distribution of PHB fragments among dividing daughter cells (Figure 6C). The lack of complete PHB consumption provides a new perspective on the physiological function of PHB. One hypothesis is that PHB may serve as an intracellular pool of reduction equivalents. Accordingly, the degradation of PHB would only be necessary under conditions of insufficient generation of reduction equivalents. However, growth at laboratory conditions usually provides a surplus of reduction equivalents, making the degradation of PHB potentially less relevant. Further studies are necessary to reveal the physiological function of PHB and the role of a putative depolymerase.

### Absence of Slr0058 Causes Growth Deficiency and More PHB Granules

Bioinformatic analysis predicts structural similarities between Slr0058 and the regulator PhaF, suggesting its involvement in the PHB metabolism (Figure 1A). Like other phasins, it is expected to be located at the granule surface and thereby influence the formation or degradation of PHB.

A knock-out mutant Δ*slr0058* shows impaired growth compared to the WT during various conditions of vegetative growth (Figure 2), as well as during chlorosis (Supplementary Figure S2A). This phenotype is merely caused by the absence of Slr0058, since a complementation restores the WT phenotype (Figure 8). This impaired growth is accompanied with a change in the absorption spectrum to a more bluish or yellowish color during vegetative growth or chlorosis, respectively (Figures 3, 5). This effect can clearly be linked to the synthesis of PHB, since a PHB synthase lacking double mutant Δ*slr0058-*Δ*PhaEC* is rescued from the phenotypes and behaves like the WT (Figure 5). Although chemical analytics clearly detected PHB in vegetatively growing Δ*slr0058* cell, no visible PHB granules could be detected. However, under appropriate conditions resulting in 2% PHB/CDW of the WT a substantial portion of the cells would contain visible granules. Since this is not the case here, PHB might be present in a non-aggregated or mis-aggregated form, which is not stained by Nile red and therefore not visible by fluorescence microscopy. The not correctly aggregated form of PHB is putatively harmful for the cells, resulting in the observed growth disadvantage of strain Δ*slr0058*, which can be rescued by preventing PHB synthesis. The synthesis of a higher residual amount of PHB in vegetatively growing Δ*slr0058* cells further implies that this protein is involved in the control of PHB synthesis.

One of the most striking features of the Δ*slr0058* strain is the greatly increased number of PHB granules during chlorosis (Figures 4C,D). This hints toward an involvement of Slr0058 in the regulation of PHB granule formation. Surprisingly, in chlorotic cells, Slr0058 seems not to be located directly at the surface of the granules (Figure 9). Instead, it aggregates in foci during vegetative growth, which slowly disappear during the course of chlorosis. The formation of GFP-Slr0058 aggregates corresponds to *in vitro* properties of purified Slr0058 protein, where the protein was mostly present in the form of dimers and trimers (Supplementary Figure S5). Possibly, the Slr0058 structures are involved in the initiation of PHB granule formation. In their absence, PHB is not properly aggregated and upon initiation of excess PHB synthesis, for example during nitrogen depletion, PHB starts to aggregate in an uncontrolled manner in numerous granules.

Polyhydroxybutyrate granules are hydrophobic structures. The surface-volume ratio of the PHB granules, and thereby the surface exposed to the cytoplasm, increases with decreasing granule size. This could lead to unintended interactions with intracellular structures, like the thylakoid membranes, resulting in a growth delay as well as a change in the pigmentation composition (Figure 3A). This requires a strict control of the surface-volume ratio. We have previously characterized the first cyanobacterial phasin, PhaP, which is essential for controlling the surface-volume ratio of PHB granules (Hauf et al., 2015). There, we have demonstrated that a lack of the phasin PhaP led to a lower average number of PHB granules per cell. Apparently, in the absence of PhaP, the cells reduce the PHB surface to volume ratio by producing less, but bigger granules. As it is known from other bacteria, PHB granules are complex and highly regulated organelles, and hence sometimes referred to as carbonosomes (Jendrossek, 2009). In other organisms, like for example *Cupriavidus necator*, many different proteins are involved in the exact formation and regulation of these organelles. It seems likely that also cyanobacteria have a strict regulation of PHB granules, where Slr0058 could play an important role for the initiation and size of such granules.

Although the physiological importance of PHB remains unclear (Damrow et al., 2016), the findings of this study increase our knowledge about PHB synthesis in cyanobacteria. A better understanding of the complex process of PHB granule formation is a step forward toward a sustainable production of biodegradable plastics from phototrophic organisms.

## Data Availability Statement

All datasets generated for this study are included in the article/Supplementary Material.

## Author Contributions

MK and KF conceptualized the study, reviewed and edited the manuscript and were responsible for the project administration. TO, JA, MK, and KF contributed to methodology. TO, JA, and MK carried out the investigation. MK, TO, and KF prepared the original draft. KF supervised the study.

## Conflict of Interest

The authors declare that the research was conducted in the absence of any commercial or financial relationships that could be construed as a potential conflict of interest.
