# Supplementary material for: The Slr0058 Protein From Synechocystis sp. PCC 6803 Is a Novel Regulatory Protein Involved in PHB Granule Formation
Source: Front Microbiol. 2020 Apr 30;11:809. doi: 10.3389/fmicb.2020.00809 (PMC7203880; doi:10.3389/fmicb.2020.00809)
Supplement: Supplementary file 1 [file Data_Sheet_1.docx]

**Table A1** – List of used bacterial strains

| Organism | Strain | Genotype | Purpose |
| --- | --- | --- | --- |
| *E. coli* | Top10 | F- *mcrA* Δ(*mrr-hsd*RMS-*mcr*BC) Φ80*lac*ZΔM15 Δ*lac*X74 *rec*A1 *ara*D139 Δ( *araleu*)7697 *gal*U *gal*K *rps*L (StrR) *end*A1 *nup*G | Molecular Cloning |
| *E. coli* | NEB10β | Δ(ara-leu) 7697 *araD139* *fhuA ΔlacX74 galK16 galE15 e14-* Φ80d*lacZ*Δ*M15* *recA1 relA1 endA1 nupG rpsL* (StrR) *rph spoT1 Δ(mrr-hsdRMS-mcrBC)* | Molecular Cloning |
| *Synechocystis* sp. PCC 6803 | wild type glucose tolerant, Tübingen | WT | Background strain, control |
| *Synechocystis* sp. PCC 6803 | Δ*slr0058* | *slr0058::Spec^R^* | Characterization of *slr0058* KO mutants |
| *Synechocystis* sp. PCC 6803 | Δ*slr0058*Δ*phaEC* | *slr0058::Spec^R^ phaEC::Kan^R^* | Characterization of *slr0058* KO and PhaEC KO mutants |
| *Synechocystis* sp. PCC 6803 | *Δslr0058_*pMK23 | *slr0058::Spec^R^ phaEC::Kan^R^ – pMK23* | Complementation of *slr0058* KO by introduction of slr0058 |
| *Synechocystis* sp. PCC 6803 | *Δslr0058_*pMK25 | *slr0058::Spec^R^ phaEC::Kan^R^ – pMK25* | Complementation of *slr0058* KO by introduction of C-terminal eGfp-tagged Slr0058 |
| *Synechocystis* sp. PCC 6803 | Δ*slr0060* | *slr0060::Cm^R^* | Characterization of *slr0060* KO mutants |

**Table A2** – List of used oligonucleotides. Underlined sequences = overhangs for Gibson primers

| #Nr | Oligo (LabNr) | Sequence (5’-3’) | Purpose |
| --- | --- | --- | --- |
| #1 | 1_pMK16_US_fw | GACCATGATTACGCCAAGCTTGCATGCCTGCAGGTCGACTACAATTGCACGATAGAGTTTATG | Gibson fragment of upstream region of *slr0060* (pMK16) |
| #2 | 2_pMK16_US_rev | GGATTTATTTATTCTGCGAATTGGTTTTGCTGCGATTAATTTG | Gibson fragment of upstream region of *slr0060* (pMK16) |
| #3 | 3_pMK16_CMR_fw | CAAATTAATCGCAGCAAAACCAATTCGCAGAATAAATAAATCCTGG | Gibson fragment of chloramphenicol resistance cassette (pMK16) |
| #4 | 4_pMK16_CMR_rev | CACCCTCACCGTCTGTGGAGGAGTTTGTAGAAACGCAAAAAG | Gibson fragment of chloramphenicol resistance cassette (pMK16) |
| #5 | 5_pMK16_slrPro_fw | TTTTGCGTTTCTACAAACTCCTCCACAGACGGTGAGGGTG | Gibson fragment of native *slr0058* promoter (pMK16) |
| #6 | 6_pMK16_slrPro_rev | TCATATTTTTTGCTCATAATTCGTGAACGTTTTAAATTTTCAGTTCTGG | Gibson fragment of native *slr0058* promoter (pMK16) |
| #7 | 7_pMK16_DS_fw | GAAAATTTAAAACGTTCACGAATTATGAGCAAAAAATATGAGC | Gibson fragment of downstream region of *slr0060* (pMK16) |
| #8 | 8_pMK16_DS_rev | AAACGACGGCCAGTGAATTCGAGCTCGGTACCCGGGGATCTCTGTCAAAGTAACCCTCAC | Gibson fragment of downstream region of *slr0060* (pMK16) |
| #9 | 9_slr0058_fw | CTCCCACAGGAGAGGGAAAG | Colony PCR for *slr0058* detection |
| #10 | 10_slr0058_rev | TGGGCCAAATTGGTCAACAG | Colony PCR for *slr0058* detection |
| #11 | 11_seq_pUC19_fwd | CTTCCGGCTCGTATGTTGTG | pUC19 Sequencing primer |
| #12 | 12_seq_pUC19_rev | GGCGATTAAGTTGGGTAACG | pUC19 Sequencing primer |
| #13 | 13_seq_cmr_cassette_pMK16 | AGAATAAATAAATCCTGGTGTC | Sequencing primer CmR cassette 1 |
| #14 | 14_seq_cmr_cassette_pMK16 | GCCGTTTGTGATGGCTTCCATG | Sequencing primer CmR cassette 2 |
| #15 | 15_pMK17_US_fwd | GACCATGATTACGCCAAGCTTGCATGCCTGCAGGTCGACTGTGTCAAATCTTTAAGGTCTGG | Gibson fragment of upstream region of *slr0058* (pMK17) |
| #16 | 16_pMK17_US_rev | GCTGCGTTCGGTCAAGAGCTCTCGTGAACGTTTTAAATTTTC | Gibson fragment of upstream region of *slr0058* (pMK17) |
| #17 | 17_pMK17_SpecRt1t2_fwd | GAAAATTTAAAACGTTCACGAGAGCTCTTGACCGAAC | Gibson fragment of spectinomycin resistance cassette (pMK17) |
| #18 | 18_pMK17_Spect1t2_rev | CACCCTCACCGTCTGTGGAGGAGTTTGTAGAAACGCAAAAAG | Gibson fragment of spectinomycin resistance cassette (pMK17) |
| #19 | 19_pMK17_proslr0058_fwd | CTTTTTGCGTTTCTACAAACTCCTCCACAGACGGTGAGG | Gibson fragment of native *slr0058* promoter (pMK17) |
| #20 | 20_pMK17_proslr0058_rev | GCCAATGGGCCCCACCCAGGGTCGTGAACGTTTTAAATTTTC | Gibson fragment of native *slr0058* promoter (pMK17) |
| #21 | 21_pMK17_DS_fwd | GAAAATTTAAAACGTTCACGACCCTGGGTGGGGCCCATTG | Gibson fragment of downstream region of *slr0058* (pMK17) |
| #22 | 22_pMK17_DS_rev | GACGGCCAGTGAATTCGAGCTCGGTACCCGGGGATCGTGCCAGGAAATTTTTGTAAAAC | Gibson fragment of downstream region of *slr0058* (pMK17) |
| #23 | 23_seq_SpecR | CGCCATCTCGAACCGACGTTGC | Sequencing primer SpecR cassette |
| #24 | 24_seq_t1t2 | CATGCCGAACTCAGAAGTGAAACG | Sequencing primer SpecR cassette/t1t2 terminator |
| #25 | 25_seq_slr0060_fwd | GTGGGCGAACTTGGTTAATG | colony for *slr0060* and sequencing primer for pMK16 |
| #26 | 26_seq_slr0060_rev | TATGGGCTAGTTTGGGTGAG | colony for *slr0060* and sequencing primer for pMK16 |
| #44 | 46_322op_oopt_fwd | CAACGCTCGGTTGCCGCCGGGCGTTTTTGCGTAATGCTCTGCCAGTGTTAC | Opening of pVZ322 and integration of oop terminator |
| #45 | 47_pVZ322_open_rev | TGCAGGAGCAGAAGAGCATACATC | Opening of pVZ322 |
| #46 | 48_gib_slr0058p_fwd | CCTGGCTTTGCTTCCAGATGTATGCTCTTCTGCTCCTGCACTCCACAGACGGTGAGGG | Forward primer for Gibson fragment of p*slr0058*_*slr0058* (pMK23 and pMK25) |
| #52 | 54_gib_slr0058_GS_linker_rev | GCTTCCGCTTCCGCTTCCGCTTCCGCTTCCACGCCAACGCTGATTTTCCAG | Reverse primer for Gibson fragment of *slr0058* + GS linker overhang |
| #53 | 55_gib_eGFP_GS_linker_fwd | GGAAGCGGAAGCGGAAGCGGAAGCGGAAGCATGAGTAAAGGAGAAGAACTTTTCACTG | Forward primer for Gibson fragment of eGFP (pMK25) + GS linker overhang |
| #54 | 56_gib_eGFP_N_rev | AAAAACGCCCGGCGGCAACCGAGCGTTGTGACTTGACGGTTATTTGTATAGTTCATCCATGCCATGTGT | Reverse primer for Gibson fragment of eGFP (pMK25) |
| #55 | 57_pVZ322_seq_fwd | CGGGCAAGTACGACATCACC | Forward colony PCR and Sequencing primer for pVZ322 inserts |
| #56 | 58_pVZ322_seq_rev | CTTGCCATCCTATGGAACTG | reverse colony PCR and Sequencing primer for pVZ322 inserts |
| #57 | 59_eGFP_seq_rev | TCGAAAGGGCAGATTGTG | Sequencing primer for eGFP |
| #58 | 60_eGFP_seq_fwd | AATTGGCGATGGCCCTGTC | Sequencing primer for eGFP |
| #59 | 61_s58_seq_rev | CAACTCGTCCATTTCCGACTG | Sequencing primer for *slr0058* in pMK25 |
| #60 | pET15b-0058 fwd | CCCTCTAGAAATAATTTTGTTTAACTTTAAGAAGGAGATATACCATGGTTTTTGCTATGGTTGAC | Forward Gibson primer for cloning of pET15b-slr0058-His |
| #61 | pET15b-0058 rev | GGGGTTATGCTAGTTAGTGGTGATGATGATGATGATGATGACGCCAACGCTGATTTTCCAG | reverse Gibson primer for cloning of pET15b-slr0058-His |
| #62 | C-8His fw | TTGGCTGCTGCCACCGCTGAGCAATAACTAGCATAACCCCTTGGGGC | Forward primer for opening Plasmid and integrate C-terminal 8-His-tag (instead of N-terminal 6-His-tag) |
| #63 | C-8His rev | GCTGCTGCCCATGGTATATCTCC | reverse primer for opening Plasmid and integrate C-terminal 8-His-tag (instead of N-terminal 6-His-tag) |
| #64 | 22 5'-pET | CCGCGAAATTAATACGACTCAC | Fwd sequencing Primer |
| #65 | 23 3-pET | CCTCAAGACCCGTTTAGAGG | Rev sequencing primer |

**Table A3** – List of used plasmids

| Plasmid | Backbone | Purpose | Reference |
| --- | --- | --- | --- |
| pUC19 | - | Backbone | (Norrander et al. 1983) |
| pVZ322 | - | Backbone | (Zinchenko et al. 1999) |
| pMK16 | pUC19 | Deletion of *slr0060* in *Synechocystis* sp. PCC 6803 | This study |
| pMK17 | pUC19 | Deletion of *slr0058* in *Synechocystis* sp. PCC 6803 | This study |
| pMK23 | pVZ322 | Complementation of Δ*slr0058* | This study |
| pMK25 | pVZ322 | Complementation of Δ*slr0058* + C-terminal eGFP tag + GS linker for localization of Slr0058 | This study |
| pJET-slr1829-30KO | pJET | Knock-out of *phaEC* in *Synechocystis* sp. PCC 6803 | Constructed by Schlebusch 2011. |
| pET15b-slr0058-His-Tag | pET15b | Overexpression plasmid for Slr0058 in *E. coli* | This study |

Supplementary figures


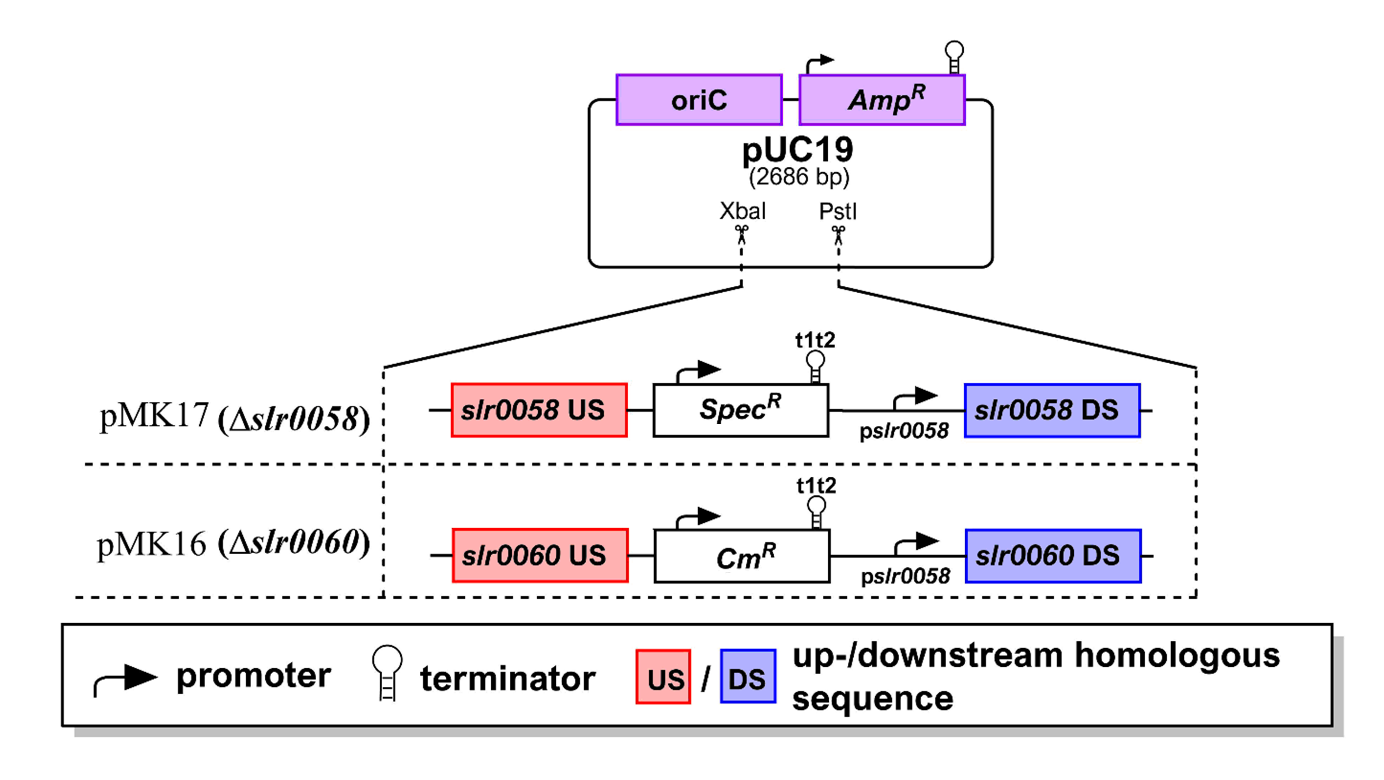


Figure S1. Structure of the plasmids pMK17 and pMK16 for the deletion of *slr0058* and *slr0060*. p*slr0058* = native promotor of *slr0058*.


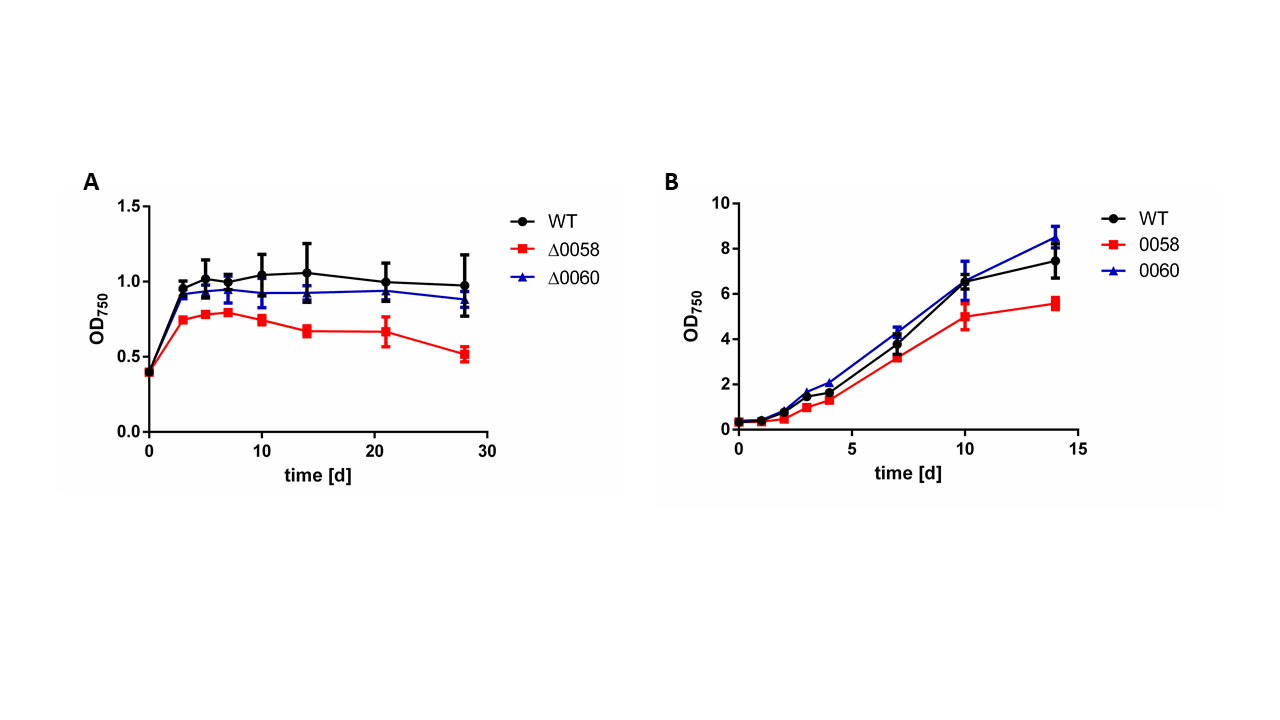


Figure S2. Cell density determined by OD_750_ after a shift to nitrogen free medium BG11_0_ (A) or after the initiation of the resuscitation (B). Each point represents a mean of three independent biological replicates.


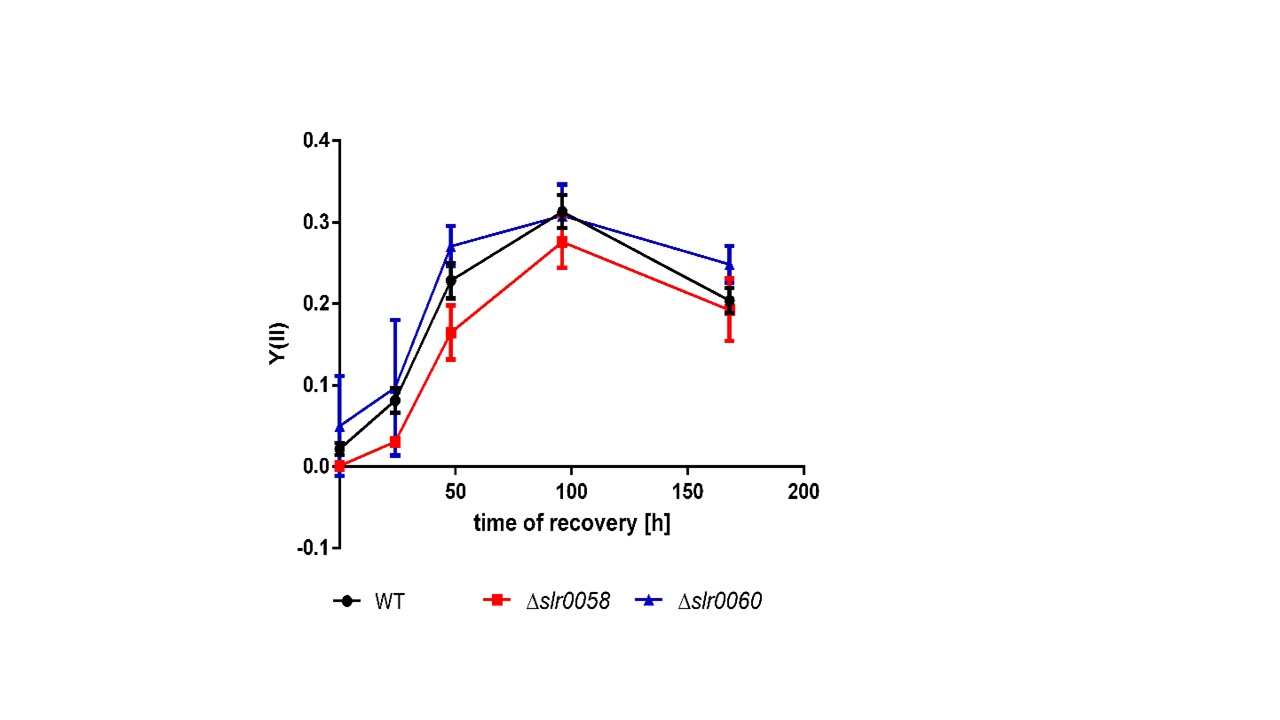


Figure S3. Yield of photosystem II (Y(II)) after initiation of the recovery. The cells used were starved from nitrogen for 14 days. Each point represents a mean of three independent biological replicates.


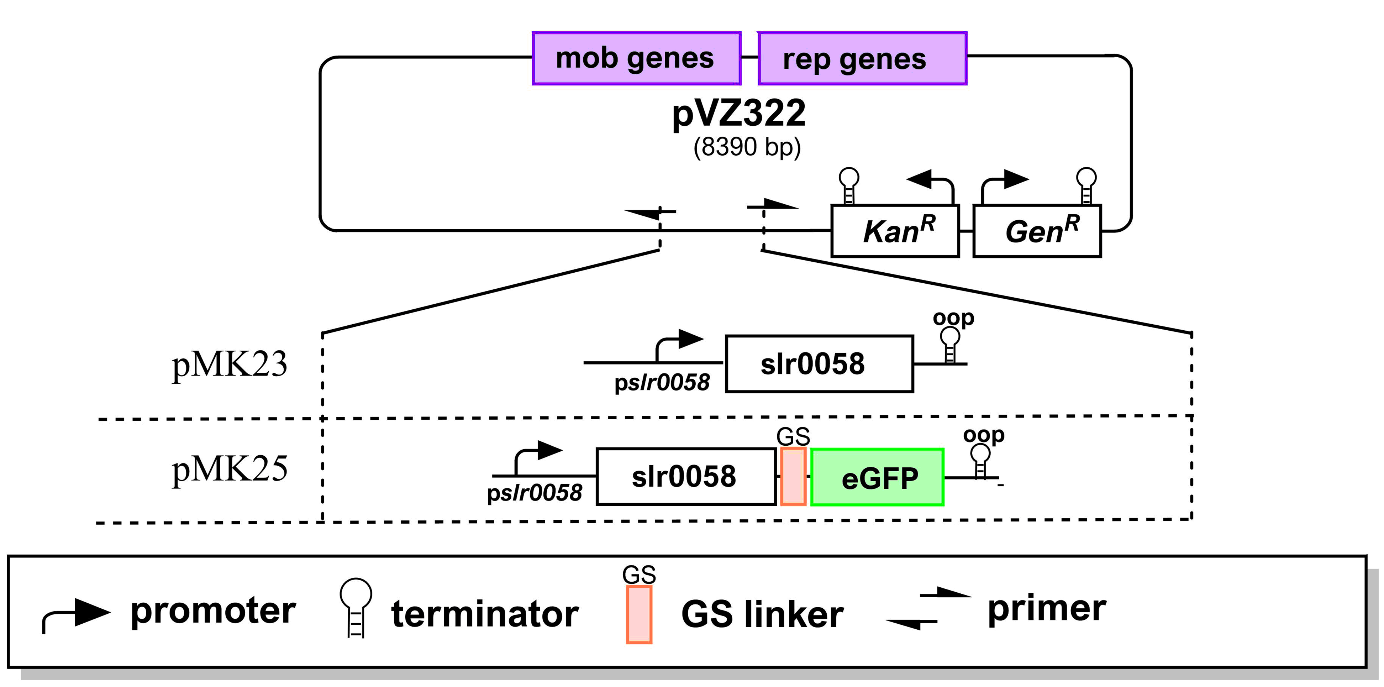


FigureS4. Structure of the plasmids pMK23 and pMK25. pMK23 was designed for the complementation of Δ*slr0058*. pMK25 was designed to express an N-terminal eGFP-tagged Slr0058 protein for localization of Slr0058 in Δ*slr0058*. For both plasmids, the genes are expressed under the natural promotor of *slr0058*, pslr0058. oop = oop terminator; mob = mobilization; rep = replication.


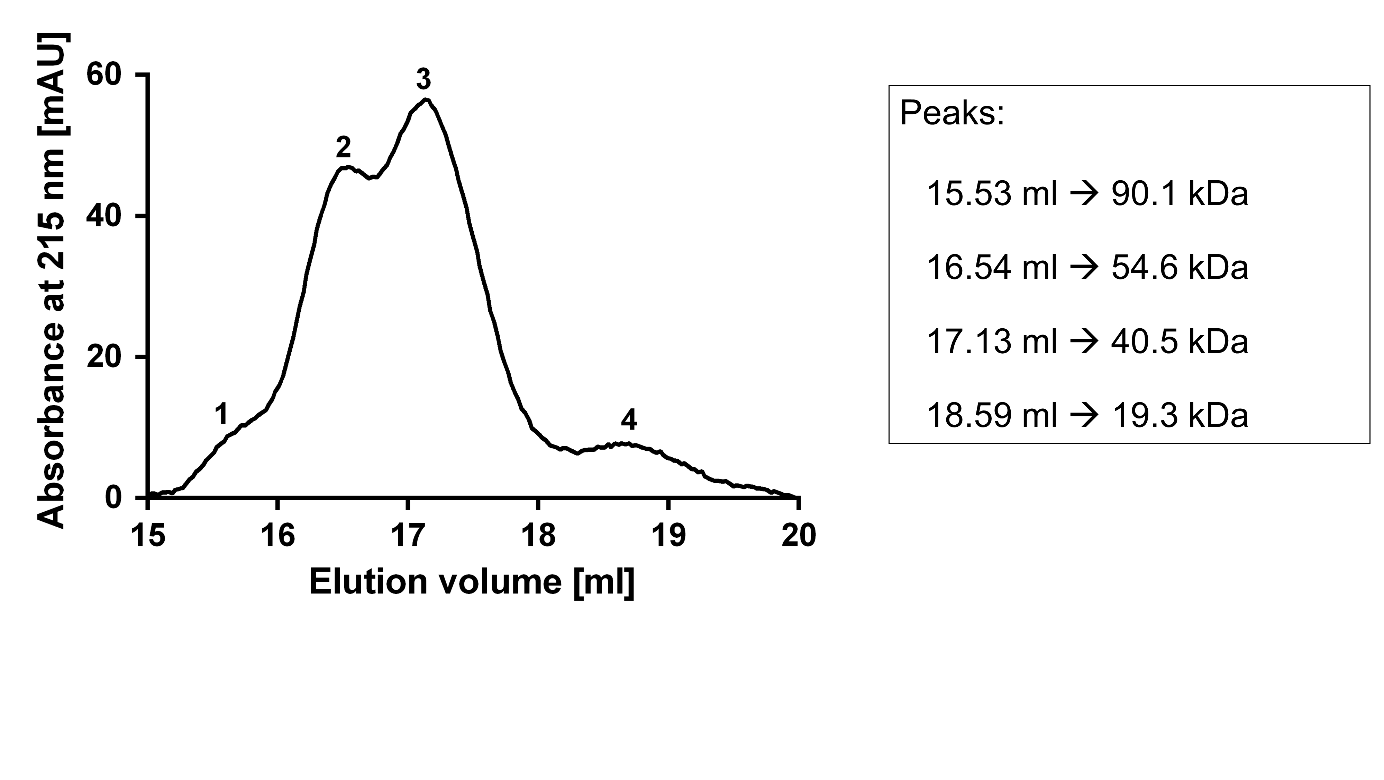


Figure S5. Analysis of oligomerization states. Overexpressed and purified Slr0058 was applied to a size exclusion column. The detected peaks and the corresponding molecular masses are shown in the table. The correlation between molecular mass and elution volume is based on a calibration curve with proteins of known sizes. The monomeric state of Slr0058 with attached His-tag has a calculated mass of 17.58 kDa.
